# Socioeconomic analysis of glaucoma patients regarding treatment options

**DOI:** 10.22336/rjo.2024.70

**Published:** 2024

**Authors:** Adin Mahmuljin, Aida Pidro Gadzo, Ajla Pidro Miokovic, Selma Hasimbegovic, Amela Dzubur Alic

**Affiliations:** 1 Eye Department at University Clinical Center Sarajevo, Sarajevo, Bosnia and Herzegovina; 2 Eye Department at “Dr. Abdulah Nakaš” General Hospital, Sarajevo, Bosnia and Herzegovina; 3 Polyclinic Vukas, Zagreb, Croatia; 4 Department for Social Medicine, School of Medicine, Univesity of Sarajevo, Sarajevo, Bosnia and Herzegovina

**Keywords:** glaucoma, life quality, treatment, socioeconomic status, Nd: YAG = neodymium-doped yttrium aluminum garnet

## Abstract

**Objective:**

To present a socioeconomic analysis of glaucoma patients regarding glaucoma treatment options.

**Methods:**

This is a prospective, comparative cross-sectional study. One hundred twenty glaucoma patients were divided into three groups. In group one, 40 patients were treated with topical therapy; group two consisted of 40 patients who had previously undergone laser therapy for glaucoma treatment; and group three had 40 patients who had undergone glaucoma surgical therapy. Data were collected using questionnaires. The data obtained by the survey were entered into the database and analyzed in the statistical program “SPSS”. The difference between the examined groups was analyzed using the “Mann-Whitney test”, and the statistical significance of the difference was analyzed using the “Hi-square test”.

**Results:**

Out of 120 patients, 65 were females, and 56 were males with a mean age of 55,6 ± 11,9 years with a range of 24-83 years. Based on the type of treatment, on average, the youngest were patients in group 2 (51.2 ± 8.3 years, range 31-66 years), followed by group 3 (55.2 ± 12.6 years, range 29-82 years), and the oldest were patients in group 1 (60.3 ± 12.6 years, range 24-83 years). Statistical analysis using the “Man-Whitney U” test showed that there was a statistically significant difference in the average age by type of treatment (p < 0.05). Other socioeconomic factors did not show statistically significant differences among groups.

**Discussion:**

Our study highlighted the interplay between socioeconomic factors and treatment choices in glaucoma management. Younger, more informed patients were more likely to opt for newer therapies like laser treatment, while older patients often relied on traditional methods. Identifying these patterns is crucial for tailoring screening programs, optimizing treatment protocols, and improving overall patient outcomes.

**Conclusion:**

Our study demonstrates few socioeconomic differences between patients in different types of glaucoma treatment.

## Introduction

A modern approach to treating glaucoma patients has shifted from intraocular pressure (IOP) control to providing the best quality of life. This way, we turn treatment and evaluation in clinical practice toward the patient as a person, not only toward the carrier of the disease. It is impossible to separate disease from personal and social contexts. One way to objectify these contexts is to assess the quality of life through different dimensions and their socioeconomic status [1].

In the U.S., about 10 million visits to ophthalmologists annually account for glaucoma-related checkups, and an average of 5.6 million prescriptions are issued for medications to treat glaucoma patients. The average cost of glaucoma treatment ranges from $ 623 per year for patients with early-stage glaucoma to $ 2,511 per year for patients with later-stage disease [2].

In our country, there are no studies or statistical data on the socioeconomics of glaucoma. Still, this disease has devastating effects on the vision and socioeconomic status of the patients, and by that, the effects on society. In this study, we analyzed the sociotechnical status of patients undergoing three different types of treatment for glaucoma.

## Methods

This was a prospective, comparative, cross-sectional, randomized study on 120 patients previously diagnosed with glaucoma from January 2011 to August 2013 in two major centers in Sarajevo. Patients were divided into three groups. The first group consisted of patients diagnosed with increased intraocular pressure with damage to the optic nerve (glaucoma) and/or the presence of specific visual field defects and who were treated with medication (40 patients); the second group consisted of patients who underwent surgery to correct elevated ocular pressure (40 patients), and the third group consisted of patients who underwent laser correction of intraocular pressure (40 patients).

The study covered both sexes equally. After the diagnosis and verification of the disease, the duration of the glaucoma was a minimum of 1 year and a maximum of 5 years.

The criteria for inclusion in the study were verified elevated intraocular pressure and/or visual field loss and/or optic nerve damage. Patients who took medication for high eye pressure (and had surgery or laser treatment for glaucoma) for not less than 1 year and not more than 5 years voluntarily consented to participate in the research. Diagnosis of glaucoma includes measuring intraocular pressure with a tonometer, detecting changes in the size and shape of the eye, examining the angle of the anterior chamber with a gonioscope, examining the optic nerve to detect possible visible damage to the nerve, and examining the visual field [3,4].

Classical surgical methods of glaucoma treatment include canaloplasty and trabeculotomy as the most common methods of conventional surgical treatment. Laser surgical treatment methods are laser trabeculoplasty, Nd: YAG laser peripheral iridotomy, and diode laser cycloablation [5].

In this research, a survey questionnaire was designed, which was intended for self-completion.

The risk factors investigated were age, gender, hereditary factors, occupation (in the past and present), the existence of myopia or hyperopia, and tobacco smoking.

### Statistical data processing

All data obtained by the survey were entered into the database and analyzed using the statistical program “SPSS”. The difference between the examined groups was analyzed using the “Mann-Whitney test”, and the statistical significance of the difference was analyzed using the “Hi-square test”. A multivariate logistic regression analysis model was used to test the correlation of variables by controlling the influence of other variables. A difference in significance level p <0.05 was considered significant.

### The ethical aspect of research

Our research has considered all the principles of the Helsinki Declaration concerning human rights. Respondents were given written instructions and consent forms to participate in the research. None of the respondents was persuaded to join the study. The participants were interviewed according to the latest methodology and instructions of the World Health Organization, with complete protection of all professional-scientific data, especially the participants’ data.

## Results

The study was conducted on 120 glaucoma patients (65 female and 56 male) with a mean age of 55,6 ± 11,9 years and a range of 24-83 years. They were divided into three groups: Group 1 consisted of patients treated with topical therapy, group 2 consisted of patients treated with laser therapy, and group 3 contained patients treated surgically.

Comparing sex and the type of treatment, group 1 consisted of 70% women, while laser therapy (55%) and surgery groups (55%) recorded a higher number of male patients. Statistical analysis by Chi-square test showed a statistically significant difference in gender distribution by type of treatment (p < 0.05).

Age structure analysis showed that the most significant number of patients in all three groups was 50-64 years. Group 1 consisted mainly of patients over 65 years (37.5% of the total number of people over 65 years); in group 2, most people were aged 35-49 (37.5% of the total number of people aged 35-49 years), and in group 3 we found the that most were younger than 35 years of age (10% of all patients treated with surgical methods were younger than 35 years of age). Analysis of age structure by age groups about the type of treatment showed statistically significant differences p < 0.05.

Based on the type of treatment, on average, the youngest were patients in group 2 (51.2 ± 8.3 years, range 31-66 years), followed by group 3 (55.2 ± 12.6 years, range 29-82 years), and the oldest were patients in group 1 (60.3 ± 12.6 years, range 24-83 years). Statistical analysis using the “Man-Whitney U” test showed a statistically significant difference in the average age by type of treatment (p < 0.05). Nevertheless, the comparison of age by sex within individual groups by type of treatment showed that no statistically significant difference was observed within any of the three observed groups, indicating the homogeneity of the sample by age concerning gender within each of the observed groups.

The analysis of marital status and type of treatment did not show statistically significant differences (p > 0.05), with the largest number of respondents in all groups being married.

Concerning the number of years of education, in all three observed groups, the highest presence was of patients with secondary education or length of schooling from 8 to 12 years and just a slightly higher number of patients with primary school in group 1. Also, there was a somewhat higher number of patients with more than 12 years of education in group 2. Statistical analysis showed no significant difference between groups according to the level of education (p > 0.05).

Analysis of patients’ occupations among groups showed that the unemployed and retirees were slightly more represented in group 1 compared to the other two groups. A significantly higher number of employees engaged in intellectual work and industrial workers were in group 3 compared to the different groups. Other occupations were generally evenly distributed within the observed three types of treatment, so statistical analysis indicated no significant difference (p > 0.05).

The total monthly income in all groups averaged 1078.7 ± 669.3 KM (range 250-4000 KM). The lowest average monthly income was in group one - 929.5 ± 623.3 KM (range 250-3000), followed by patients in group 3 - 1149 ± 557.5 KM (range 500-2600 KM), and the highest average monthly income was recorded in group 2 - 1157.5 ± 795.7 KM (range 380-4000 KM). Given the large ranges and high standard deviation values, no statistically significant difference in monthly income was observed between the groups (p > 0.05).

Chronic disease was recorded in almost identical percentages in all three groups, with the highest rate in group 3 - 60%, 55% of patients undergoing laser treatment, and 57.5% of patients undergoing topical treatment. Accordingly, statistical analysis indicated the absence of a statistically significant difference (p > 0.05). The most common chronic disease in all three groups of 64 patients who had chronic disease was hypertension, followed by diabetes mellitus and rheumatoid arthritis. Statistical analysis via Chi-squares with Yates correction showed no statistically significant difference between groups according to the type of chronic disease (p > 0.05). The mean duration of chronic disease in group 1 was 21.7 ± 12.3 years (range 5-40 years), in group 2 - 14.9 ± 14.1 years (range 3-60 years), and in group 3 - 17.4 ± 12.5 years (range 5-50 years). The duration of chronic disease did not show a statistically significant difference based on Man-Whitney rank analysis (p > 0.05).

Cigarette smoking as a risk factor did not show a statistically significant difference between groups (p > 0.05). Analysis of the correlation between the duration of the disease and tobacco consumption did not show a statistically significant correlation (p > 0.05). However, it was moving in a positive direction, which indicated that patients who smoked longer had a slightly longer duration of the disease. The disease duration differed statistically significantly according to the type of treatment (p < 0.05). Thus, patients in group 2 had the shortest average disease duration of 7.4 ± 5.1 years (range 1-21 years), while both other groups had almost twice the duration of the disease (**Table 1**).

**Table 1:** Duration of the disease in groups

	N	X	SD	SG	Minimum	Maximum
**Group I**	40	12,05	8,978	1,420	1	35
**Group II**	40	7,43	5,114	,809	1	21
**Group III**	40	12,30	9,395	1,485	2	40
**Total**	120	10,59	8,306	,758	1	40

N - number of patients, X - mean, SD - standard deviation, SG - standard error, Group I - subjects treated with topical therapy, Group II - subjects treated with laser, Group III - subjects treated surgically

Glaucoma in the family among groups and genders showed statistically significant differences between genders only in group 1 (p <0.05). In contrast, no statistically significant difference was observed in other groups or the total sample.

Analysis of other eye diseases by type of treatment did not show a statistically significant difference (p < 0.05), as shown in **Table 2**. The lowest percentage of other eye diseases was found in group 1 (12.5%), followed by group 2 (15%), and the highest rate of different eye diseases (27.5%) in group 3. In 25 patients who had other eye diseases, there was a statistically significant difference among groups (p < 0.05). The most important number of cataracts was observed in group 3. Therefore, combined surgery was performed. No significant difference was observed between other diseases and glaucoma in the groups.

**Table 2:** Presence of other eye diseases in groups

			Group			Total
			Group I	Group II	Group III	
**Other eye disease**	Cataract	N	2	0	11	13
		%	20,0	0,0	81,8	45,5
	Trauma	N	1	2	1	4
		%	20,0	33,3	9,1	18,2
	RD	N	1	2	0	3
		%	20,0	33,3	0,0	13,6
	DR	N	2	2	1	5
		%	40,0	33,3	9,1	22,7
**Total**		N	6	6	13	25
		%	100,0	100,0	100,0	100,0

N - number of patients, % - percentage, Group I - subjects treated with topical therapy, Group II - subjects treated with laser, Group III - subjects treated surgically, RD - Retinal detachment, DR - diabetic retinopathy

The analysis of diopters by type of treatment showed that 82.5% of topically treated patients had diopters, followed by 62.5% of laser-treated patients and 57.5% of surgically treated patients. Statistical analysis showed a statistically significant difference between groups according to the presence of diopter (p < 0.05).

Length of wearing glasses showed that patients in group 1, on average, wore glasses for the longest time at 23.5 ± 17.8 years (range 2-60 years), followed by group 3 - 23.3 ± 9.1 years (range 10-30 years), and group 2 - 14.5 ± 8.4 years (range 3-40 years). Statistical analysis indicated a significant difference in the duration of wearing glasses according to the types of treatment (p < 0.05).

## Discussion

With its social and economic consequences, blindness represents a significant burden on any society and has a greater effect on developing countries, older people, and women [6]. As the population ages, glaucoma becomes the second most common cause of blindness in the world after cataracts. Globally, 57.5 million people (95% CI 46.4 to 73.1 million) were affected by POAG in 2015, rising to 65.5 million (95% CrI 52.8, 83.2 million) by 2020 [7].

Based on the results of our study, we could discuss the impact of the disease treatment on the socioeconomic status of examined patients and use these data to plan for future screening programs and treatment protocols.

In our study, patients treated with medication in 70% of cases were women, while in the other two groups, we recorded a higher number of male patients (55% in both groups). Other studies state that women are more represented in all types of glaucoma, so if we compare this with our research, we can conclude that we obtained the same results even though we had slightly more represented male patients in laser and surgical treatments [8]. The higher number of male patients might be because male patients are more persistent in a search for more aggressive treatment options in our country.

Analysis of the average age of the subjects in our study showed that the average age of the total sample was 55.6 years, which coincided with other studies that underline that glaucoma most often affects people older than 45 [9].

Statistical analysis using the “Man-Whitney U” test showed a statistically significant difference in the average age according to the type of treatment between groups (p < 0.05), and the youngest subjects who underwent laser treatment (51.2 ± 8.3 years) ranged from 31-66 years. Since the laser method is the latest treatment for elevated intraocular pressure in glaucoma, the average age of those treated with this method was lower than other groups. Similar results were obtained by researchers at the Wills Eye Institute in Philadelphia in the United States in 2012 [10].

The analysis of marital status, years of education, occupations of patients, and their total monthly income by type of treatment did not show statistically significant differences that would indicate their impact on more frequent or rare cases of glaucoma. Research worldwide shows that the quality of life of glaucoma patients is negatively affected by lower socioeconomic status, lower level of education, and lack of information about the nature of the disease because such people are usually not familiar with the best way to treat the disease [8].

Chronic disease was recorded in almost identical percentages in all three groups, with the highest rate in the group undergoing surgical treatment - 60%, in 55% of patients. Accordingly, the statistical analysis indicated the absence of a statistically significant difference, i.e., the presence of the chronic disease did not affect the choice of therapeutic procedure for the treatment of glaucoma, according to our study.

The most common chronic disease in all three groups of 64 patients who had chronic disease was hypertension, followed by diabetes mellitus and rheumatoid arthritis. Statistical analysis via Chi-squares with Yates correction for cases of tables with cell values less than 5 showed no statistically significant difference between groups according to the type of chronic disease (p > 0.05). Other studies have also found that hypertension is the most common eye disease associated with glaucoma. Still, the data did not show an association of an eye disease that would influence the choice of glaucoma therapy [11].

The analysis of the presence of cigarette smoking as a risk factor did not show statistically significant differences both between genders within individual groups and between individual groups (p > 0.05). Many studies have investigated the impact of smoking on the development of glaucoma in the last 30 years, with different results. The first research conducted in the 1970s showed that smoking one pack of cigarettes a day can lead to an increase in the intraocular pressure of up to 5 mmHg [12], while recent research shows the impact of nicotine on water production in terms of increased production. Therefore, cigarette smoking is described as an additional factor for the development of glaucoma [13].

The duration of the disease was directly related to age, with a statistically significant difference between age groups (p < 0.05). Thus, the youngest patients had the shortest duration of the disease of 6.6 ± 9.9 years (range 1-29), and patients older than 65 years had the most extended duration of 22.8 ± 8.6 years (range 7-40 years). The disease duration was statistically significantly different according to the type of treatment (p < 0.05). Thus, patients treated with laser had the shortest average disease duration of 7.4 ± 5.1 years (range 1-21 years), while patients treated with surgery and medication had almost twice the disease duration. This might be related to the fact that younger patients are better informed about new treatment methods than older patients, who have used therapy to lower intraocular pressure for a long time and have not changed it.

The analysis of the presence of glaucoma in the family by sex and groups showed that a statistically significant difference between the sexes existed only in the group of patients undergoing drug treatment (p < 0.05). In contrast, no statistically significant difference was observed in other groups or the total sample. The presence of glaucoma in the family increases the risk of glaucoma by 4-9 times [14].

By analyzing the presence of other eye diseases, we observed no statistically significant differences in gender, both according to individual types of treatment and in the total sample (p > 0.05).

We observed that in the group treated surgically, most patients were present with cataracts, and they had a combination of cataract surgery and glaucoma surgery. In the other groups, no significant difference was observed between the presence of different diseases and glaucoma. In a total of 22 patients, a statistically significant difference was observed (x2 = 13.116; p = 0.041). Patients treated surgically also reported the presence of another eye disease (cataract).

After surgical cataract treatment, postoperative reduction of intraocular pressure usually occurs, but with elevated intraocular pressure, which is not reduced to drug therapy, cataract surgery and trabeculectomy can be performed in one surgical act. Research worldwide shows that in patients older than 65 years, with the onset and development of cataracts, there is often an increase in intraocular pressure, and a combined cataract surgery and trabeculectomy or iridectomy are practiced [15].

The analysis of the length of wearing glasses (diopters) showed that patients treated with medication on average wore glasses for the longest time at 23.5 ± 17.8 years (range 2-60 years), followed by surgically treated patients at 23.3 ± 9.1 years (range 10-30 years) and laser-treated patients at 14.5 ± 8.4 years (range 3-40 years). Statistical analysis indicated a significant difference in the duration of wearing glasses according to the types of treatment (p < 0.05). Research worldwide shows the association of myopia as a risk factor for glaucoma. At the same time, the occurrence of presbyopia cannot be linked to glaucoma except in the sense that age as a risk factor and the occurrence of presbyopia after age 45 are related [16].

Our study demonstrated few socioeconomic differences between patients in different types of glaucoma treatment. Younger patients tend to be more informed and prone to react faster to the shift to laser or surgery therapy [17]. Investigating many other aspects and the quality of life of all groups would be attractive. Reaction to treatment was different and individual, and in most cases, it took some time to find the best possible therapeutic option for each patient. Their socioeconomic status guided us toward prompt therapeutic decisions.

## Conclusions

Based on the results of our study, we could explore the significant impact of glaucoma treatment on patients' socioeconomic status, particularly highlighting how different treatment modalities influence their quality of life, financial burden, and access to care. These findings underscore the importance of tailoring treatment approaches to individual patient needs while addressing educational barriers, socioeconomic conditions, and access to advanced therapies. Furthermore, this data provides valuable insights for developing targeted screening programs and optimizing treatment protocols to enhance patient outcomes, reduce disease progression, and mitigate the societal burden of glaucoma. Such efforts are essential for improving early detection and ensuring equitable access to effective treatment, particularly in underserved populations.
